# The Growth Performance and Nutrient Composition of Black Soldier Fly (*Hermetia illucens*) Larvae Fed Slaughtered Bovine Blood

**DOI:** 10.3390/insects15090635

**Published:** 2024-08-25

**Authors:** Hao Bian, Yuting Qiao, Yantong Li, Zifan Wang, Lei Zhao, Zhiqiang Li, Bo Cheng, Gongtao Ding

**Affiliations:** 1 Key Laboratory of Biotechnology and Bioengineering of State Ethnic Affairs Commission, Biomedical Research Center, Northwest Minzu University, Lanzhou 730030, China; b18735763157@163.com (H.B.); liyantong0623@163.com (Y.L.); zifanwang0316@163.com (Z.W.); zhaolei@xbmu.edu.cn (L.Z.); 2 School of Life Sciences, Lanzhou University, Lanzhou 730000, China; qiaoyt21@lzu.edu.cn; 3 School of Medicine, Northwest Minzu University, Lanzhou 730030, China; yxlzq@xbmu.edu.cn

**Keywords:** slaughter blood, kitchen waste, black soldier fly larvae

## Abstract

**Simple Summary:**

The disposal of slaughterhouse blood poses significant environmental challenges due to its biological instability and high nutrient content. This study investigates the potential of using black soldier fly larvae (BSFL) to process slaughtered cattle blood mixed with kitchen waste. We examined the growth performance and nutrient composition of BSFL when fed different proportions of slaughtered blood. The results revealed that feeding BSFL with high levels of slaughtered blood is not suitable, as it leads to high mortality rates and poor growth. However, when fed with up to 20% slaughtered blood combined with kitchen waste, BSFL showed excellent performance in terms of weight gain and crude protein, lipid, and amino acid contents. This demonstrates that slaughtered blood can be effectively used as a feed component for BSFL, enabling the recovery of essential nutrients and reducing their loss within the production system. Our findings highlight the potential of this approach for sustainable waste management and animal feed production, offering an environmentally friendly solution to the disposal of slaughterhouse blood.

**Abstract:**

The disposal of slaughterhouse blood poses significant environmental challenges due to its biological instability and high nutrient content. We used a gradient of 10% blood increments (0–100%) to feed BSFL, and the correlation between the proportion of bovine blood and the BSFL weight gain, mortality rate, fatty acid content, and amino acid content was researched. Results indicate a positive correlation between the bovine blood content and BSFL mortality, with survival rates above 95% for blood proportions below 60%. Larval weight exhibited a negative correlation as the bovine blood content increased. Nutritional analysis revealed that the crude protein content in BSFL increased proportionally with bovine blood (14.75–25.45 g/100 g), while the crude fat content decreased correspondingly (10.70–4.66 g/100 g). The sugar content remained relatively constant across groups. Fatty acid analysis showed increased levels of C16:0, C14:0, and C16:1 and decreased levels of C18:1, C18:2, and C18:3 with higher bovine blood contents. The amino acid content generally increased with higher blood proportions. This study highlights the bioconversion potential of BSFL for bovine blood and underscores the impact of protein, lipid, and sugar concentrations in feed on BSFL growth. These findings provide valuable insights for utilizing slaughterhouse waste in BSFL rearing, contributing to the development of more sustainable waste management and animal feed production methods.

## 1. Introduction

**Figure 1:**
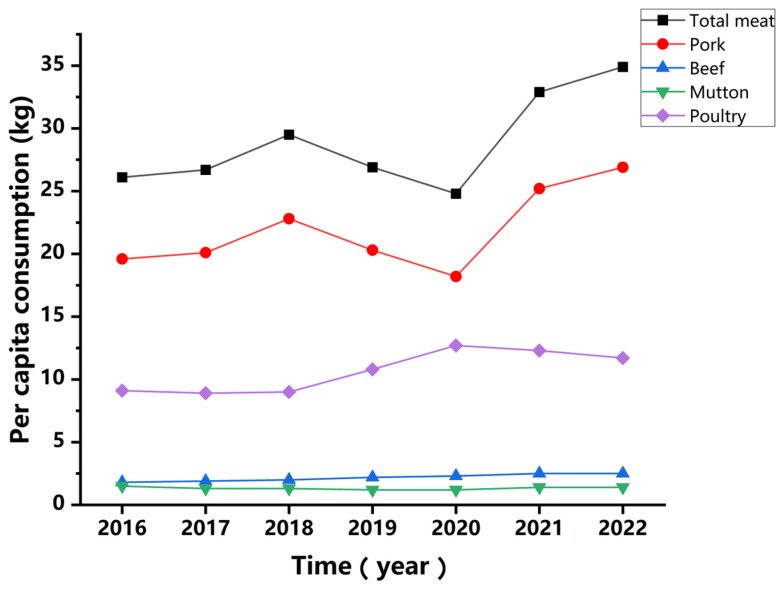
Per capita meat consumption in China from 2016 to 2022.

Disposing of blood from slaughterhouses poses a significant environmental challenge in meat production, necessitating effective management solutions [1]. Slaughterhouse blood’s high nutritional and moisture contents lead to biological instability, including biodegradation, enzymatic activity, and the potential spread of pathogens, making its management particularly complex [2,3]. In China, which has a high per capita meat consumption, the volume of livestock slaughter is substantial (Figure 1). In 2023, China produced 7.53 million tons of beef, 5.31 million tons of mutton, 57.94 million tons of pork, and 25.63 million tons of poultry meat. Based on a blood-to-body weight ratio of 3.5%, approximately 3.37 million tons of waste blood was generated from pigs, cattle, sheep, and poultry slaughter in 2023. The inefficient utilization of these resources creates a negative feedback loop, impacting environmental sustainability, food security, and public health. Therefore, developing technically feasible, economically viable, and environmentally friendly approaches for food and feed production and organic waste treatment is crucial.

China has relatively comprehensive treatment plans for slaughtered animal offal, making slaughter blood the main slaughter waste [4]. Slaughter blood is primarily used to produce blood meal for animal feed or as a compound fertilizer for plant growth. However, this process requires significant energy and chemical input for transportation, storage, and treatment [5]. In recent years, the immense quantity of blood from cattle, pigs, sheep, and poultry slaughter has not been effectively treated and utilized, leading to its discharge into the environment and resulting in substantial resource waste [6]. Slaughterhouses use a large amount of water for hygiene during processing operations, generating a large amount of wastewater mixed with a significant amount of suspended solids and liquid waste, such as blood, fat, feces, urine, and tissues. This waste can contaminate surface water and groundwater, leading to odor issues, soil pollution, and other related environmental problems [7]. Blood is one of the primary dissolved pollutants in slaughterhouse wastewater, with an average chemical oxygen demand (COD) of 8000 mg/L, the highest in slaughterhouse wastewater [8]. If the blood from a cow carcass can be discharged directly into the sewer, the sewage load would be equivalent to the total daily sewage generated by 50 people [8].

Given the concerns of greenhouse gas emissions, environmental pollution, and the potential for using organic and agricultural waste as feed, insect rearing is deemed a sustainable production route [9]. The black soldier fly (*Hermetia illucens*), belonging to the Stratiomyidae family and the *Hermetia* genus, is distributed worldwide in subtropical and tropical regions [10]. Black soldier fly larvae (BSFL) are widely used for the resource utilization of organic waste [11]. BSFL are considered a promising biotechnology for harmless waste treatment and resource utilization due to their broad feeding range and ability to convert organic waste efficiently [12]. The conversion and degradation of waste by BSFL reduce the environmental hazards of waste, minimize resource waste, and promote the recovery of organic matter within the BSFL [13]. The treatment of organic matter by BSFL has been successfully applied in livestock farming, aquaculture, and other industries [14,15,16]. The environmentally friendly treatment of blood is costly and continues to rise, increasing meat production costs. The sales price of carcasses alone cannot sufficiently offset the high capital investment required for raising animals. Therefore, finding methods to utilize by-products, such as blood, can boost profits, sustain the meat industry, and mitigate issues related to their disposal.

Currently, there are only a few studies on feeding BSFL slaughter blood and investigating their growth and nutrient performance. We propose using slaughter blood to feed BSFL to address the issues of slaughter blood resource utilization and BSFL feed material sources. This study explores the feasibility of using slaughter blood as a feeding substrate for BSFL and the effect of feed containing different proportions of slaughter blood on the protein content, lipid content, fatty acid profile, and amino acid profile of BSFL.

## 2. Materials and Methods

### 2.1. BSFL Incubation

BSFL were obtained from the China-Malaysia National Joint Laboratory, Northwest Minzu University, China. The 0-day-old larvae were fed a diet composed of chicken feed, bran, rice husks, and water (1:2:2:15, *v*/*v*) and reared for 4 days under controlled conditions (20 ± 5% RH and 28 ± 3 °C). The BSFL were then screened using a nylon mesh with a grid size of 1.5 mm × 1.5 mm. Live BSFL weighing less than 20 mg could pass through the mesh into the container, while feed, dead larvae, and larger larvae were blocked. The container was then inverted and shaken clockwise to remove minor impurities and larvae. The final weight of the BSFL ranged from 4.5 mg to 20 mg.

### 2.2. Larvae Feeding

Eleven feeding substrates with different mixing ratios were prepared and tested for larval feeding in the experiment. The substrates were used to feed the same cohort of BSFL. The substrates were grouped according to the mixing ratio (*w*:*w*) of slaughter blood and food waste, with gradients of 10%, totaling 11 groups(Table 1). The mixed substrates were thoroughly crushed and evenly stirred. Each experiment is performed in triplicate.

Each substrate group was fed to 50 randomly selected screened BSFL for 3 days to investigate mortality rates and determine the acceptable mixture ratio of slaughter blood and kitchen waste for feeding. 

The feedable substrates were SF1–SF7. Each group contained 2000 larvae, reared in boxes measuring 30 cm × 20 cm × 5 cm, with a density of 0.3 cm²/larva, under 20 ± 5% RH and 28 ± 3 °C, in continuous darkness. Fresh substrate (200 g) was fed daily, and 50 BSFL were randomly selected, weighed, and recorded daily over a 10-day feeding cycle. Following the feeding trial, all larvae were collected and quickly frozen with liquid nitrogen. The weight gain of BSFL was modeled using the logistic equation to establish the weight growth kinetics of each group, described by the logistic differential equation:

(1) $$\frac{dX}{dt}=\mu_{max}\left(1-\frac{X}{X_{max}}\right)X$$

*X*: BSFL weight (mg); *μ_max_*: maximum specific growth rate (d^−1^); *X_max_*: maximum BSFL weight (mg); *t*: feeding time (d); When *t* = 0, *X* = *X*_0_; *X*_0_: initial BSFL weight (mg). Integrating Equation (1) gives Equation (2):

(2) $$X\left(t\right)=\frac{X_{max}}{1+e^{2-\mu_{max}t}}$$

### 2.3. Component Analysis

#### 2.3.1. Total Lipid Content Detection

The extraction of lipids was performed using the Soxhlet extraction method. The apparatus employed was a BUCHI Soxhlet Extractor B-811 (Flawil, Canton of St. Gallen, Switzerland), with petroleum ether (boiling range 30–60 °C) as the extraction solvent [17]. In brief, 1 g of each sample was weighed and placed in an evaporating dish. Approximately 20 g of quartz sand was added, and the mixture was evaporated to dryness on a boiling water bath. Subsequently, the sample was dried in an electric forced-air drying oven at 100 °C ± 5 °C for 30 min. After cooling, the sample was finely ground and transferred entirely into a filter-paper thimble. The evaporating dish and any glass rod with adhering sample were cleaned using ether-soaked, defatted cotton, which was then placed into the thimble. The thimble was inserted into the extraction chamber of the Soxhlet apparatus, which was connected to a pre-weighed receiving flask. Petroleum ether was added through the top of the condenser until it filled approximately two-thirds of the flask’s volume. The apparatus was heated on a water bath, allowing the petroleum ether to continuously reflux and extract (6–8 cycles per hour) for 6–10 h. The extraction was deemed complete when a drop of the extract left no oily residue on a ground glass rod. Upon completion, the receiving flask was removed, and the petroleum ether was recovered. When only 1–2 mL of solvent remained in the flask, it was evaporated on a water bath. The flask was then dried at 100 °C ± 5 °C for 1 h, cooled in a desiccator for 0.5 h, and weighed. This process was repeated until a constant weight was achieved (defined as two consecutive weightings differing by no more than 2 mg).

#### 2.3.2. Total Protein and Moisture Content Detection

The total protein content of the samples was determined using the Kjeldahl method [18]. Each semi-solid sample was weighed into a digestion tube. A Kjeldahl tablet and 20 mL of concentrated sulfuric acid (ρ = 1.84 g/mL) were added, and the mixture was subjected to digestion in a digestion furnace. Once the temperature of the digestion furnace reached 420 °C, the digestion process was continued for an additional hour. After cooling, 50 mL of water was added to the digested sample. The subsequent processes of liquid addition, distillation, and titration were performed automatically using an automatic Kjeldahl nitrogen analyzer (Foss Analytical, Hillerød, Capital Region of Denmark, Denmark), and the titration data were recorded. The protein content in the sample was calculated according to Equation (3):

(3) $$X=\frac{\left(V_{1}-V_{2}\right)\times c\times 0.014}{m\times V_{3}÷100}\times F\times 100$$

*X*: the content of protein in the sample (g/100 g); *V*_1_: the volume of sulfuric acid standard titration solution consumed by the test solution (mL); *V*_2_: the volume of sulfuric acid standard titration solution consumed by the reagent blank (mL); *c*: the concentration of sulfuric acid standard titration solution (mol/L); *m*: the mass of the sample (g); *V*_3_: the volume of digestion solution taken (mL); *F*: nitrogen to protein conversion factor (Larvae, *F* = 4.76; The feeding substrate, *F* = 6.25).

The SF1–SF7 matrices and each group of BSFL samples were heated at 100 °C ± 5 °C until dry, cooled, and then weighed. This process was repeated until a constant weight was achieved, and the final weight was recorded; the moisture content of the sample can be ultimately determined.

#### 2.3.3. Total Sugar Content Detection

One gram of each sample (accurate to 0.01 g) was weighed into a 250 mL Erlenmeyer flask. To this, 25 mL of water and 10 mL of hydrochloric acid were added. The mixture was agitated to form a homogeneous suspension. A reflux condenser was attached, and the sample was hydrolyzed in a boiling water bath for 1 h. After cooling, the mixture was filtered, and the residue was washed. The filtrate and washings were combined and diluted to a final volume of 250 mL. This solution was then homogenized and reserved for analysis as the test solution. An aliquot of 1 mL of the test solution was accurately pipetted into a 10 mL colorimetric tube. To this, 1 mL of phenol solution (50 g/L) was added, followed by 5 mL of concentrated sulfuric acid (ρ = 1.84 g/mL). The reaction mixture was allowed to stand for 10 min, after which it was vortexed and cooled to room temperature. The absorbance was measured at a wavelength of 490 nm, with the blank solution used to zero the instrument and measure the absorbance of the sample [19]. This method can be used to detect the total content of monosaccharides, simple oligosaccharides, and some easily hydrolyzable polysaccharides in the sample (not including chitin).

#### 2.3.4. Analysis of the Fatty Acid Composition of BSFL

To 100 mg of lipid extract, 8 mL of 2% sodium hydroxide in methanol solution was added. The mixture was connected to a reflux condenser and refluxed in a water bath at 80 °C ± 1 °C until oil droplets disappeared. Subsequently, 7 mL of 15% boron trifluoride in methanol solution was added through the top of the reflux condenser, and the reflux was continued for an additional 2 min at 80 °C ± 1 °C. The reflux condenser was rinsed with a small amount of water. Heating was then discontinued, and the flask was removed from the water bath and rapidly cooled to room temperature. Precisely 10–30 mL of n-heptane was added, and the mixture was shaken for 2 min. Saturated sodium chloride solution was then added, and the mixture was allowed to separate into layers. Approximately 5 mL of the upper n-heptane extract was transferred to a 25 mL test tube. To this, 3–5 g of anhydrous sodium sulfate was added, and the mixture was shaken for 1 min and then left to stand for 5 min. The upper layer was then transferred to a sample vial for analysis. The analysis was performed using an Agilent 7000D Triple Quadrupole GC/MS system (Santa Clara, CA, USA) equipped with a DB-wax column (30 m × 250 μm × 0.25 μm). The chromatographic conditions were as follows: injector temperature, 240 °C; carrier gas flow rate, 2 mL/min; initial column temperature, 140 °C held for 5 min, then ramped to 180 °C at 4 °C/min and held for 1 min, followed by ramping to 200 °C at 2 °C/min and held for 2 min, then to 220 °C at 2 °C/min and held for 2 min, and finally to 230 °C at 5 °C/min and held for 5 min. The injection volume was 1 μL, and the split mode was employed with a split ratio of 20:1. The separated compounds enter the mass spectrometer and are identified based on their mass-to-charge ratio. Mass spectra and retention time peaks can be used to qualitatively identify different compounds. Quantitative analysis of FAME in a sample can be performed by comparing the chromatographic peak areas or peak heights of standards of known concentrations [20].

#### 2.3.5. Analysis of Amino Acid Composition of BSFL

The protein or polypeptide sample was subjected to acid hydrolysis to release its constituent amino acids. For the detection of Cys and Met, the sample was first protected with formic acid (at twice the volume of the sample) prior to acid hydrolysis, and then, the sample was treated with 6 M hydrochloric acid at a high temperature (110 °C) for 12 h. A 100 μL aliquot of the sample solution was taken and added to 100 μL of 0.1 M NaHCO_3_ buffer (pH 9.0), followed by the addition of 20 μL of fluorenylmethyloxycarbonyl chloride (Fmoc-Cl) solution. The mixture was vortexed and allowed to react at room temperature for 5 min. The reaction was then quenched by adding 100 μL of 0.1 M phosphate buffer (pH 2.5). The derivatized sample was filtered through a 0.22 μm membrane filter and injected into a high-performance liquid chromatography (HPLC) system for the analysis of 17 hydrolyzed amino acids. The injection was performed with an Agilent 1260 Infinity Autosampler (Santa Clara, CA, USA) at a flow rate of 1 mL/min. Gradient elution was employed using a mobile phase composed of solvent A (0.1% trifluoroacetic acid in water) and solvent B (0.1% TFA in acetonitrile), starting from 90:10 (A:B) and gradually increasing the proportion of solvent B to 50:50 over 30 min. The analysis was conducted on a C18 reverse-phase column (Agilent ZORBAX Eclipse Plus C18, 4.6 × 150 mm, 5 μm) (Santa Clara, CA, USA). Fluorescence detection was performed with an excitation wavelength of 265 nm and an emission wavelength of 340 nm. All samples were dissolved in the mobile phase, and all measurements were carried out at ambient temperature [21].

### 2.4. Statistical Processing

Statistical processing was performed using GraphPad Prism 9 (GraphPad Software, San Diego, CA, USA) for experimental data and graphing. One-Way ANOVA was employed to determine significant differences among different groups of larvae reared with varying ratios of slaughter blood and kitchen waste. Tukey’s Honestly Significant Difference (HSD) post-hoc test was used to assess significant differences, with significance set at *p* < 0.001. All results were expressed as the mean ± SD.

## 3. Results

### 3.1. BSFL Growth Performance

**Figure 2:**
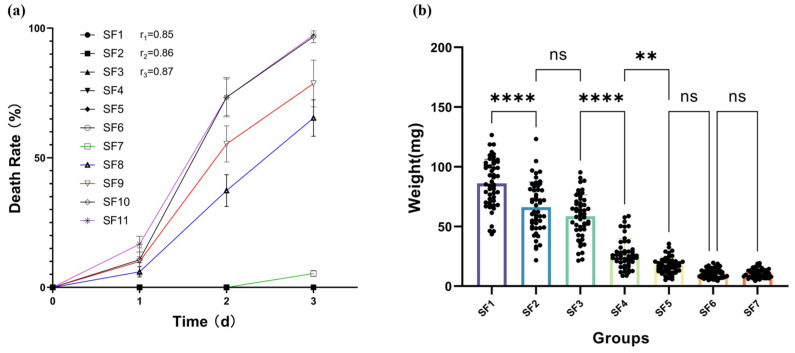
Effects of different levels of feeding substrates on BSFL: (**a**) mortality effects of SF1–SF11 substrates on BSFL; (**b**) One-Way ANOVA analysis of BSFL weight on the tenth day. r, the Pearson correlation coefficient between the mortality rate of BSFL and the content of slaughter blood in the feed during a three-day trial. Asterisks indicate significant differences between groups: ns = not significant (*p* > 0.05), ** *p* < 0.01, **** *p* < 0.0001.

Different substrates showed varying results in BSFL growth, most notably in weight gain. Survival rates differed among substrates, with higher levels of slaughter blood showing lower survival rates. During the 3-day trial, there was no significant impact on BSFL survival rates in the lower slaughter blood level groups SF1–SF7, with survival rates above 95%. The higher slaughter blood level groups SF8–SF11 had over 65% mortality rates (Figure 2a). Thus, BSFL survival rates were negatively correlated with the blood content in the feed. Subsequent trials excluded SF8–SF11 due to their higher mortality rates.

Differential analysis showed that BSFL weights in groups with different blood contents mainly clustered into four groups: SF1, SF2–SF3, SF4, and SF5–SF7. There were no significant differences between any two groups within each cluster. SF1’s final weight was significantly higher than the others, and although SF2–SF3 significantly differed from SF1, they were much higher than SF4–SF7. Weight analysis categorized them into three clusters: SF1, SF2–SF3, and SF4–SF7. Compared to SF1, the smallest difference in SF2–SF3 (SF3/SF1 = 68.05%) was much higher than the largest in SF4–SF7 (SF4/SF1 = 20.07%) (Figure 2b). Therefore, BSFL weight was negatively correlated with blood content in the feed.

### 3.2. Growth Kinetics of BSFL

**Figure 3:**
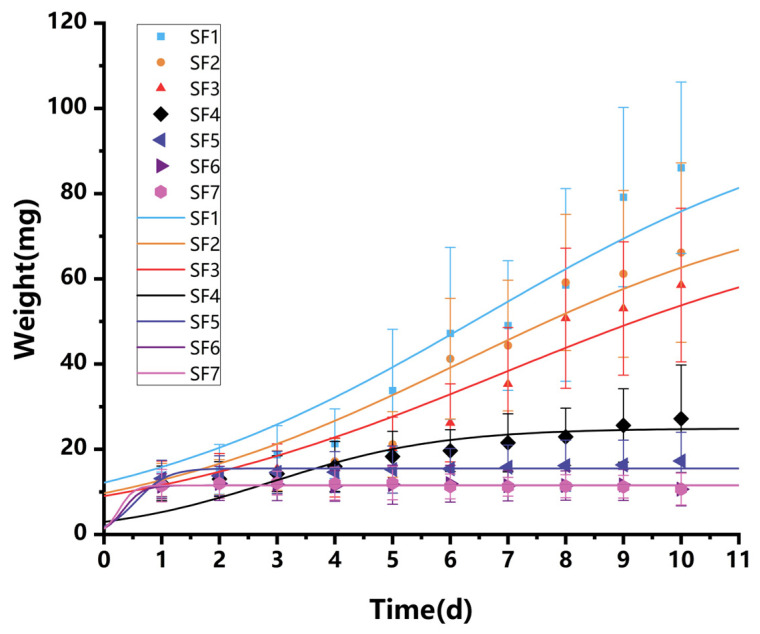
Kinetic analysis of BSFL growth.

In the 10-day feeding trial, substrates with less than 20% slaughter blood content demonstrated good growth outcomes. As shown in Figure 3, the body weight gain of SF1–SF3 during the 10-day trial followed a generally S-shaped growth curve. From day 0 to 4, the BSFL were in a lag phase, characterized by slow growth and nearly flat weight gain. From day 5 to 8, they entered a rapid growth phase, with accelerated weight gain. Finally, from day 9 to 10, the growth of BSFL slowed down as they entered a plateau phase.

The growth curve of BSFL approximates an S-shaped curve, and the logistic equation is a typical S-shaped equation [22]. Therefore, the logistic equation is suitable for the growth process of BSFL. The integral form of the logistic equation is presented in Formula (2). Based on experimental data, the parameters *μ_max_* and *X_max_* for SF1–SF4 were obtained by fitting using Origin2022 (Table 2).

According to the dynamic model of BSFL weight growth during the growth process, the dynamic model fitting curve of the BSFL growth process was obtained by applying Origin2022 (Figure 3). The growth dynamics coefficients of determination (R^2^) of SF1–SF4 were 91.33%, 90.51%, 89.84%, and 59.71%, indicating that these three dynamic models can reflect the growth process of BSFL well, and the experimental data fit well. The closer the R² value is to 1, the better the model fits the data and provides more accurate predictions. The larvae in groups SF5, SF6, and SF7 showed virtually no growth, making them unsuitable for fitting the logistic model. Consequently, successful fitting could not be achieved.

### 3.3. Composition of BSFL and Feeding Substrates

**Figure 4:**
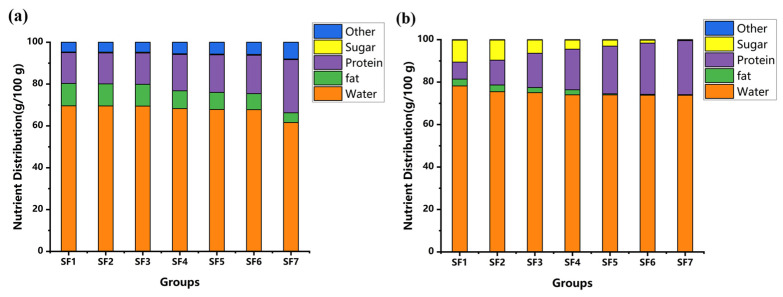
Composition results of BSFL and feeding substrate: (**a**) composition results of BSFL from SF1–SF7; (**b**) composition results of the feeding substrate from SF1–SF7. “Other” may include minerals, vitamins, and undetected polysaccharides (such as chitin).

The proportions of biochemical components of the experimental substrates and cultured BSFL are shown in Figure 4. Regarding the crude protein content of BSFL, the SF1–SF3 test groups were similar (14.75–14.95 g/100 g, *p* > 0.05), the SF4–SF6 test groups were similar (17.38–18.34 g/100 g, *p* > 0.05), and the SF7 test group was the highest (25.45 g/100 g, *p* > 0.05). The crude protein content was proportional to the slaughter blood content in the feed. The moisture content of BSFL decreased with the increase in the slaughter blood content in the feeding substrate, but the difference in moisture content was not significant (61.59–69.60 g/100 g, *p* > 0.05). The crude fat content of BSFL was similar in SF1–SF3 test groups (10.41–10.70 g/100 g, *p* > 0.05), similar in SF4–SF6 test groups (7.67–8.51 g/100 g, *p* > 0.05), and lowest in the SF7 test group (4.66 g/100 g, *p* > 0.05). The crude fat content of BSFL was inversely proportional to the slaughter blood content in the feed. Interestingly, the overall difference in the sugar content of BSFL was insignificant (0.29–0.40 g/100 g, *p* > 0.05) (Figure 4a). Regarding the feeding substrates, the crude fat content, sugar content, and moisture content were inversely proportional to the slaughter blood content in the feed, while the crude protein content was proportional to the slaughter blood content in the feed (Figure 4b).

### 3.4. Fatty Acid Composition of BSFL

Fatty acid composition analysis was performed on BSFL from experimental groups SF1 to SF7. The following fatty acids were present in all experimental groups: C12:0 (lauric acid), C14:0 (myristic acid), C16:0 (palmitic acid), C16:1 (palmitoleic acid), C18:0 (stearic acid), C18:1 (oleic acid), C18:2 (n-6) (linoleic acid), and C18:3 (n-3) (linolenic acid).

Saturated fatty acids (SFAs) were the most abundant in BSFL, exceeding 50% in each group. Among SFAs, C12:0 was the most prevalent (27.23–30.51%), followed by C16:0 (13.02–17.74%) (Table 3). 

**Figure 5:**
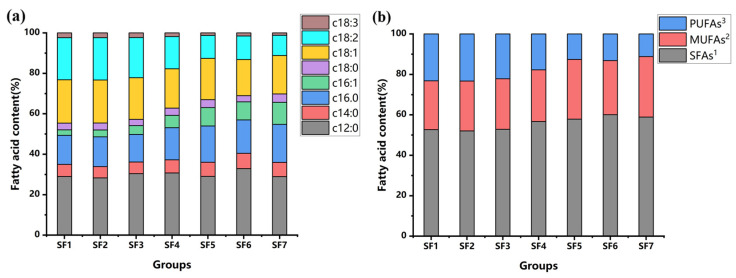
Fatty acid composition results of BSFL: (**a**) Composition results of C12:0, C14:0, C16:0, C16:1, C18:0, C18:1, C18:2 (n-6), and C18:3 (n-3); (**b**) composition results of SFA, MUFAs, and PUFAs. ^1^ Saturated fatty acids. ^2^ Monounsaturated fatty acids. ^3^ Polyunsaturated fatty acids.

The SFA content increased with increasing proportions of slaughterhouse blood in the substrate (Figure 5b). This increase was mainly attributed to the rise in C16:0 and C14:0, while C12:0 and C18:0 did not show significant changes in response to increasing slaughterhouse blood levels (Figure 5a).

Monounsaturated fatty acids (MUFAs) were primarily represented by C18:1 (16.56–20.54%). The content of C18:1 exhibited a negative correlation with the proportion of slaughterhouse blood in the substrate, while C16:1 showed a positive correlation (Figure 5a). Overall, MUFAs did not display a clear increasing or decreasing trend due to the contrasting relationship of C18:1 and C16:1 with the slaughterhouse blood content (Figure 5b).

Polyunsaturated fatty acids (PUFAs) decreased with an increasing slaughterhouse blood content (Figure 5b). C18:2 (9.48–20.15%) was the predominant PUFA. Both C18:2 and C18:3 contents showed a negative correlation with the proportion of slaughterhouse blood in the substrate (Figure 5a).

### 3.5. Amino Acid Content of BSFL

Seventeen hydrolyzed amino acids were analyzed in BSFL from feeding groups SF1 to SF7 after the feeding trial (Table 4). There were substantial differences in the total amino acid contents across the groups, with the hydrolyzed amino acid content roughly positively correlated with the slaughter blood content. Group SF3 had the lowest hydrolyzed amino acid content (732.71 nmol/mg), followed by SF1 (806.20 nmol/mg). The most abundant amino acids in all groups were alanine and glutamic acid, while cysteine was present in the lowest amounts.

**Figure 6:**
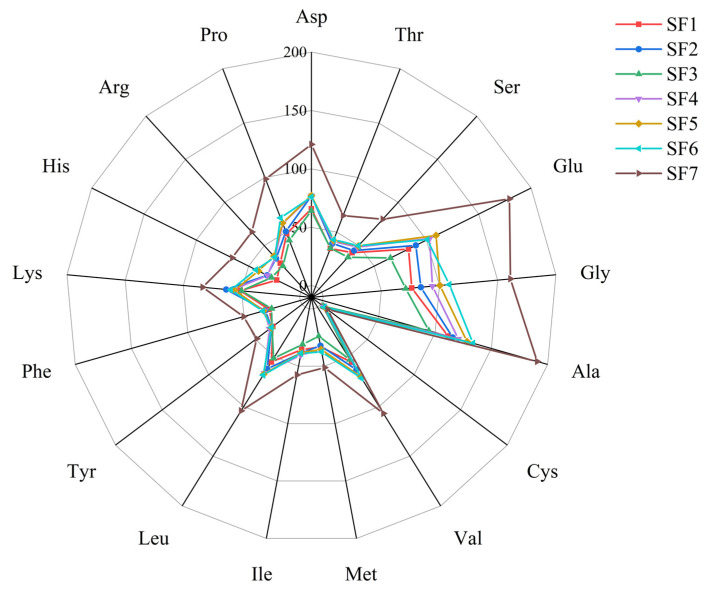
Amino acid composition results of BSFL.

As illustrated in Figure 6, except for SF3, the content of the 17 hydrolyzed amino acids in each group increased with the amount of slaughter blood added, with noticeable changes in the levels of glutamic acid (Glu), glycine (Gly), and alanine (Ala), all showing distinct boundaries on the network graph and consistent growth trends. Among the 17 hydrolyzed amino acids, the ones with the highest concentrations included aspartic acid (Asp), glutamic acid (Glu), glycine (Gly), alanine (Ala), valine (Val), and leucine (Leu). Additionally, the network graph indicated that the group with the most significant differences was SF7, in which all amino acid levels were higher than those in the remaining experimental groups.

## 4. Discussion

Insects can serve as a valuable medium for recycling essential nutrients from waste and as a value-added feed component, which is conducive to vigorously developing a circular economy, improving resource utilization efficiency, and aligning with the sustainable development goals of various countries [23,24]. This study demonstrates that feeding slaughterhouse blood to BSFL can effectively recover essential nutrients, reducing the loss of these nutrients within the production system. The ability of BSFL to reduce food waste, livestock manure, and other wastes has been widely studied [25,26,27], and our research holds significant implications for the application of BSFL in slaughterhouse blood treatment programs.

The results indicate a strong correlation between BSFL adaptability and higher substitution levels of slaughterhouse blood, leading to significant changes in growth performance and increased time required for BSFL development. These differences gradually become apparent with an increased feeding duration. This situation is similar to Rodrigues’ study [28], which analyzed this trend due to high levels of protein and lipids. In our study, BSFL growth was negatively correlated with the slaughterhouse blood concentration and positively correlated with the lipid content. BSFL are not suitable for feeding on substrates with slaughterhouse blood concentrations exceeding 60%, exhibiting high mortality at high concentrations. One possible reason for the short-term lethality could be the toxic intermediate substances produced by the decomposition of slaughterhouse blood. Toxic substances, such as total volatile elemental nitrogen (TVB-N) and biogenic amines, are produced during the putrefaction of slaughterhouse blood. There are numerous types of biogenic amines, most of which are toxic, with histamine being the most toxic, causing blood diseases and neurotoxicity. Putrescine, spermidine, cadaverine, and spermine can form carcinogenic nitrosamines with nitrites [29,30,31]. Higher concentrations of slaughterhouse blood decomposition can produce high levels of toxic substances that exceed the tolerance limit of BSFL, leading to death [31].

BSFL grow better in feed with low concentrations (≤20%) of slaughterhouse blood, while high concentrations (≥30%) lead to poor growth, so it is necessary to control the blood addition reasonably to optimize production efficiency. Considering the overall experimental results, adding 10% and 20% slaughterhouse blood concentrations to the feed can achieve optimal production efficiency. The mixed feeding substrate in this study needs to consider the recovery and treatment of slaughterhouse blood and production costs, such as BSFL growth efficiency. High slaughterhouse blood content experiments adversely affect the final larval weight, indicating that nutritional imbalances or sudden dietary changes can have negative impacts, such as poor BSFL weight gain and longer life cycles [32]. Other literature has provided different reasons to explain these variations in the life cycle when rearing BSFL on sludge, plant-based organic waste, and food waste, including low protein levels, high moisture levels, high lipid levels, and low bioavailability of the feeding substrate, all of which can cause poor BSFL weight gain and longer life cycles [33,34,35,36,37]. In this study, the moisture content (73–78 g/100 g) of each group’s substrate was optimal for BSFL [24]. However, the protein concentration gradually increased as the slaughterhouse blood concentration increased in the substrate. At the same time, lipids and sugars gradually decreased, which may be the main factors leading to poor BSFL weight gain and longer life cycles. The experimental groups with better growth were those with ≤20% slaughterhouse blood addition. Although the 10% and 20% slaughterhouse blood addition groups had increased BSFL growth cycles compared to those without slaughterhouse blood, the impact was less than half that of the 30% slaughterhouse blood addition group (Figure 2b). Additionally, the kinetic study shows that the specific growth rate of the three groups of BSFL varies with time, with the maximum specific growth rate of BSFL occurring during the transition from the lag phase to the logarithmic growth phase. In actual farming, the kinetic model derived from this experiment can be used to monitor production and substrate addition during the logarithmic growth phase of BSFL to increase the growth rate and improve production efficiency (Figure 3).

The main components of dry-weight BSFL are protein and lipids (Figure 4a), while the content of components, such as sugars, is low. The components of BSFL in each group correspond to the components of the substrate, indicating that BSFL have a high conversion rate for sugars, as sugars are degraded before proteins, leading to a higher carbohydrate degradation rate [38]. The similarity of component distribution is higher in the experimental groups with ≤20% slaughterhouse blood addition, while the similarity is higher in the experimental groups with 30–50% slaughterhouse blood addition, both showing that BSFL store fewer lipids and the profound influence of the slaughterhouse blood content on the BSFL protein content, which directly affects the distribution of fatty acids and amino acids in each group of BSFL. Starvation can lead to nutritional deficiencies in BSFL, which may result in reduced fat content and decreased body weight, consistent with our experimental results [39]. However, nutritional deficiencies typically also lead to a reduction in the protein content, which is not consistent with our findings [40]. Therefore, it remains unclear whether starvation is the underlying cause of the observed low fat content.

The primary fatty acids in BSFL in this experiment are SFAs, with the most common SFAs in BSFL being C12:0 (lauric acid), C14:0 (myristic acid), C16:0 (palmitic acid), and C18:0 (stearic acid) [41,42]. The experimental results show that the total amount of SFAs increases with the increase in the slaughterhouse blood content, mainly due to the increase in C12:0 and C16:0. However, the lipid content in the substrate gradually decreases. Hoc’s experiment explained this phenomenon, where the concentration of SFAs in the substrate was lower than that in larval tissues, indicating that the SFAs in BSFL are resynthesized [43]. Regarding monounsaturated fatty acids (MUFAs) in BSFL, C18:1 has the highest content. The content of C18:1 and polyunsaturated fatty acids (PUFAs) decreases with an increasing slaughterhouse blood concentration, which is consistent with the results confirmed by Fernando et al. that BSFL can bioaccumulate PUFAs and MUFAs [36,44]. Chen et al.’s experimental results showed that BSFL lipids and protein can improve chickens’ immunity, antioxidant functions, intestinal morphology, and barrier functions, significantly increasing chicken blood’s BUN and AST levels [45]. This suggests that BSFL lipids have a significant role in their innate immunity, thus synthesizing and storing large amounts of lipids. Feeding these BSFL to livestock and poultry can help enhance their immune functions [46]. In addition to lipids being beneficial for promoting chicken growth, BSFL also contain all the essential amino acids that promote optimal growth performance in chickens [46,47]. 

In this experiment, except for the 30% slaughterhouse blood group, the amino acid content in each group was positively correlated with the slaughterhouse blood content, and the amino acid content in BSFL may change according to the life stage or diet of BSFL [48]. The protein content of BSFL is positively correlated with the concentration of slaughter blood in the feed; therefore, we speculate that the decrease in amino acid content in the BSFL of the SF3 group is related to protein synthesis [49].

## 5. Conclusions

The results of this study demonstrate that BSFL have good biotransformation potential. Using a certain proportion of slaughtered cattle blood as a feeding substrate for BSFL can effectively recover biological resources from slaughtered cattle blood. Additionally, our research found that the composition of the feeding substrate significantly impacts BSFL growth. The experimental groups with ≤20% slaughterhouse blood addition performed better in terms of the body weight, crude protein content, lipid content, and amino acid content of the cultured BSFL. Although high levels of slaughterhouse blood can increase the crude protein and various fatty acid and amino acid contents of BSFL, it leads to slower larval weight gain and higher mortality rates, making it unsuitable for feeding BSFL. BSFL cultivation is a promising method for transforming slaughterhouse blood into value-added insect biomass for further utilization.

## Figures and Tables

**Table 1:** Composition of the feeding substrate.

Composition	Group Percentage Content (%)										
	SF1	SF2	SF3	SF4	SF5	SF6	SF7	SF8	SF9	SF10	SF11
Slaughter blood	0	10	20	30	40	50	60	70	80	90	100
Food waste	100	90	80	70	60	50	40	30	20	10	0

Trials: SF is the abbreviation for slaughter plasma and food waste. SF1–11 correspond to different mixed feeding substrates.

**Table 2:** Parameter values in the growth kinetic model.

Groups	*X_max_*	*μ_max_*	R^2^
SF1	102.06862	0.30613	91.33%
SF2	81.29864	0.32108	90.51%
SF3	75.74053	0.28956	89.84%
SF4	24.91285	0.68278	59.71%

**Table 3:** Fatty acid composition of BSFL lipid extracts. Abundance values are expressed as the mean abundance (%) (±standard deviation), and values are the average (±standard deviation) of five samples (*n* = 3). Means with different superscript letters differ significantly (*p* < 0.05).

	SF1	SF2	SF3	SF4	SF5	SF6	SF7
c12:0	27.76 ± 2.83 ^a^	27.23 ± 1.19 ^a^	29.04 ± 2.54 ^a^	28.87 ± 0.9 ^a^	27.23 ± 0.05 ^a^	30.51 ± 0.37 ^a^	27.34 ± 0.47 ^a^
c14:0	5.72 ± 0.17 ^d^	5.41 ± 0.11 ^e^	5.55 ± 0.26 ^d,e^	6.15 ± 0.04 ^c^	6.55 ± 0.04 ^b^	7.05 ± 0.09 ^a^	6.66 ± 0.21 ^b^
c16.0	13.81 ± 0.58 ^d^	14.12 ± 0.16 ^d^	13.02 ± 0.63 ^e^	14.91 ± 0.32 ^c^	16.83 ± 0.1 ^b^	15.33 ± 0.1 ^c^	17.74 ± 0.07 ^a^
c16:1	2.58 ± 0.08 ^g^	3.29 ± 0.03 ^f^	4.18 ± 0.16 ^e^	5.72 ± 0.17 ^d^	8.5 ± 0.05 ^b^	8.27 ± 0.16 ^c^	10.31 ± 0.11 ^a^
c18:0	3.17 ± 0.16 ^d^	3.32 ± 0.08 ^c,d^	2.93 ± 0.18 ^e^	3.38 ± 0.06 ^c^	3.66 ± 0.02 ^b^	2.87 ± 0.05 ^e^	3.92 ± 0.04 ^a^
c18:1	20.54 ± 1.04 ^a^	20.47 ± 0.49 ^a^	19.71 ± 1.07 ^a,b^	18.27 ± 0.22 ^c,d^	19.16 ± 0.17 ^b,c^	16.56 ± 0.26 ^e^	17.95 ± 0.4 ^d^
c18:2	19.95 ± 1.01 ^a^	20.15 ± 0.47 ^a^	18.99 ± 1 ^b^	15.03 ± 0.17 ^c^	10.63 ± 0.1 ^d^	10.86 ± 0.16 ^d^	9.48 ± 0.14 ^e^
c18:3	2.27 ± 0.12 ^a^	2.27 ± 0.06 ^a^	2.2 ± 0.12 ^a^	1.67 ± 0.02 ^b^	1.16 ± 0.02 ^e^	1.38 ± 0.02 ^d^	1.1 ± 0.02 ^e^
SFAs ^1^	50.46 ± 2.28 ^c^	50.09 ± 1.07 ^c^	50.54 ± 2.25 ^c^	53.31 ± 0.5 ^b^	54.27 ± 0.15 ^a,b^	55.76 ± 0.38 ^a^	55.66 ± 0.68 ^a^
MUFAs ^2^	23.12 ± 1.11 ^c^	23.76 ± 0.46 ^b,c^	23.89 ± 1.12 ^b,c^	23.99 ± 0.37 ^b,c^	27.67 ± 0.22 ^a^	24.84 ± 0.22 ^b^	28.25 ± 0.51 ^a^
PUFAs ^3^	22.22 ± 1.13 ^a^	22.41 ± 0.53 ^a^	21.19 ± 1.11 ^b^	16.7 ± 0.19 ^c^	11.79 ± 0.12 ^d^	12.24 ± 0.18 ^d^	10.57 ± 0.17 ^e^

^1^ Saturated fatty acids. ^2^ Monounsaturated fatty acids. ^3^ Polyunsaturated fatty acids.

**Table 4:** Contents of 17 amino acids in BSFL. Abundance values are expressed as relative abundance (nmol/mg). Means with different superscript letters differ significantly (*p* < 0.05).

	SF1	SF2	SF3	SF4	SF5	SF6	SF7
Asp	65.71 ^d^	76.88 ^b^	64.15 ^e^	76.77 ^b^	77.09 ^b^	75.93 ^c^	120.93 ^a^
Thr	34.91 ^f^	39.41 ^e^	35.18 ^f^	41.40 ^d^	42.19 ^c^	42.94 ^b^	65.07 ^a^
Ser	41.75 ^e^	44.18 ^d^	36.55 ^f^	48.15 ^c^	49.53 ^b^	49.80 ^b^	80.50 ^a^
Glu	82.72 ^f^	89.80 ^e^	65.53 ^g^	102.98 ^c^	109.02 ^b^	100.65 ^d^	179.21 ^a^
Gly	76.22 ^f^	84.04 ^e^	71.01 ^g^	94.01 ^d^	100.37 ^c^	108.45 ^b^	160.96 ^a^
Ala	111.44 ^f^	115.58 ^e^	94.49 ^g^	120.64 ^d^	128.33 ^c^	133.72 ^b^	190.68 ^a^
Cys	2.89 ^e^	3.03 ^e^	2.47 ^f^	3.72 ^d^	5.02 ^b^	4.06 ^c^	7.26 ^a^
Val	55.35 ^e^	62.94 ^d^	52.77 ^f^	69.34 ^c^	69.67 ^c^	71.53 ^b^	106.94 ^a^
Met	33.34 ^d^	32.15 ^e^	23.97 ^f^	35.38 ^c^	35.34 ^c^	37.62 ^b^	51.27 ^a^
Ile	35.44 ^e^	38.55 ^c,d^	30.95 ^f^	40.38 ^b^	38.28 ^d^	38.81 ^c^	57.54 ^a^
Leu	55.12 ^f^	61.99 ^e^	51.36 ^g^	66.27 ^d^	67.29 ^c^	68.26 ^b^	104.33 ^a^
Tyr	31.36 ^e^	32.73 ^c^	32.32 ^d^	34.93^b^	33.01 ^c^	32.82 ^c^	48.78 ^a^
Phe	27.78 ^f^	31.01 ^e^	25.41 ^g^	31.58 ^d^	32.43 ^c^	33.17 ^b^	50.29 ^a^
Lys	51.04 ^f^	63.27 ^b^	50.04 ^g^	58.34 ^d^	56.05 ^e^	59.22 ^c^	83.46 ^a^
His	23.15 ^g^	32.48 ^d^	28.07 ^f^	32.05 ^e^	40.55 ^c^	42.95 ^b^	65.72 ^a^
Arg	29.55 ^f^	34.96 ^e^	26.16 ^g^	36.12 ^d^	37.67 ^b^	36.46 ^c^	65.74 ^a^
Pro	48.43 ^f^	50.48 ^e^	42.29 ^g^	57.88 ^d^	58.35 ^c^	62.92 ^b^	98.55 ^a^
Total	806.20	893.48	732.71	949.93	980.17	999.31	1537.21

## Data Availability

Data are available upon request from the corresponding author and pending agreement by co-authors.
